# Questionable necessity effects on suicidal ideation: a commentary on Marchetti et al. (2026)

**DOI:** 10.3389/fpsyg.2026.1794113

**Published:** 2026-03-16

**Authors:** Kimmo Sorjonen, Bo Melin, Marika Melin

**Affiliations:** Department of Clinical Neuroscience, Karolinska Institutet, Stockholm, Sweden

**Keywords:** analytic rigor, mental health, necessary condition analysis (NCA), spurious effects, suicidal ideation

## Abstract

Marchetti et al. recommended researchers of mental health to use necessary condition analysis (NCA). In an empirical application, they found statistically significant necessity effects of perceived burdensomeness and thwarted belonging on suicidal ideation. However, necessity effects in NCA do not prove that X is necessary for Y, as the effect could, for example, be due to a correlation between X and Y and this correlation could, in turn, be due to a confounding impact by a third variable Z. We scrutinized the findings and conclusions by Marchetti et al. by estimating “ranges of spuriousness,” i.e., 95% confidence intervals of necessity effects that could be expected due to the correlations between suicidal ideation and perceived burdensomeness and thwarted belonging, respectively. The necessity effects fell within the range of spuriousness, meaning that they could be accounted for by the correlations between the variables. Hence, conclusions by Marchetti et al., that perceived burdensomeness and thwarted belonging are necessary for suicidal ideation, may be challenged. It is important for researchers using NCA to be aware of the method’s severe limitations. We recommend scrutinizing findings by estimating ranges of spuriousness and to require that necessity effects are above this range if concluding that X is necessary for Y.

## Necessary condition analysis (NCA)

According to a traditional definition, if X is necessary for Y, Y will never be the case if X is not the case. As an example, if being human is necessary for being Greek, all Greeks are human and not being human is sufficient for not being Greek. Necessary condition analysis (NCA) estimates the size of the empty space in the upper-left corner in a XY-plot, as a percentage of the area given by (X_*max*_ − X_*min*_) × (Y_*max*_ − Y_*min*_), and labels this percentage as a necessity effect. The size of the empty space/necessity effect is presumed to indicate to what degree a low value on X precludes a high value on Y (Dul, 2016). Statistical significance of the necessity effect can be estimated by a permutation test (Dul et al., 2020). For example, Marchetti et al. (2026) used NCA and concluded, based on statistically significant necessity effects, that perceived burdensomeness and thwarted belonging were necessary conditions for clinically relevant levels of passive suicidal ideation. This would mean that high levels of suicidal ideation were not possible with a low score on perceived burdensomeness or thwarted belonging, i.e., low scorers would, in the words of Marchetti et al., be “immune” to suicidal ideation. Although we acknowledge the programmatic and conceptual nature of the article by Marchetti et al., we believe that their conclusions may be challenged.

## Limitations and added rigor

Analyses have shown that necessity effects in NCA may be spurious consequences of a correlation between X and Y which, in turn, may be spurious due to a confounding impact by a third variable Z. Spurious statistically significant necessity effects may be observed already with a weak correlation between X and Y, especially with a large sample (Sorjonen and Melin, 2025). Moreover, the degree of bias is the same irrespective of variables measure psychological, medical, biological, sociological, or some other type of constructs. Hence, necessity effects in NCA do not prove that X is necessary for Y. For increased analytic rigor, we have proposed estimating a “range of spuriousness” by generating a large number (e.g., 1000) of datasets with the same sample size and correlation between X and Y as in the empirical data and to estimate the necessity effect in each of these generated datasets. The range of spuriousness corresponds to the 95% (or some other chosen value) confidence interval across these estimated necessity effects. If the original necessity effect falls within or below this confidence interval it is not significantly stronger than could be expected due to the correlation between X and Y. This would mean that the necessity effect can be accounted for by the correlation between X and Y and claims that X is necessary for Y should be postponed (Sorjonen and Melin, 2025).

The range of spuriousness corresponds to a control group. As an analogy, we should not necessarily claim success if individuals taking a supposedly enhancing drug can lift significantly more than zero kilos on average, as people can be expected to lift more than zero kilos even without enhancing drugs. Therefore, we should compare the performance of these individuals with a control group that has taken placebo. Similarly, we should not claim, as is the present norm, necessity as soon as a necessity effect in NCA differs significantly from zero. Instead, we should compare with what the necessity effect can be expected to be solely due to the correlation between X and Y, i.e., the range of spuriousness.

Here, we estimated ranges of spuriousness to scrutinize the findings and conclusions by Marchetti et al. Analyses were conducted with R 4.4.3 statistical software (R Core Team, 2025) using the haven (Wickham et al., 2023), MASS (Venables and Ripley, 2002), NCA (Dul, 2021), and lavaan (Rosseel, 2012) packages. The analytic script, which also downloads used data, is available at the Open Science Framework at https://osf.io/n7gsk/. The analytic script includes a function (NCArigour) that takes sample size, correlation between X and Y, and estimated necessity effect as input and delivers results as reported below. The function defines the random seed number in each run, meaning that the results reported below would be replicated if rerunning the script. Following Marchetti et al., we analyzed data used and made available by Teo et al. (2018). Also following Marchetti et al., we used the so-called ceiling envelopment with free disposal hull (CE-FDH) technique to estimate the necessity effect (Dul, 2016).

## Questionable necessity effects and an alternative model

**FIGURE 1:**
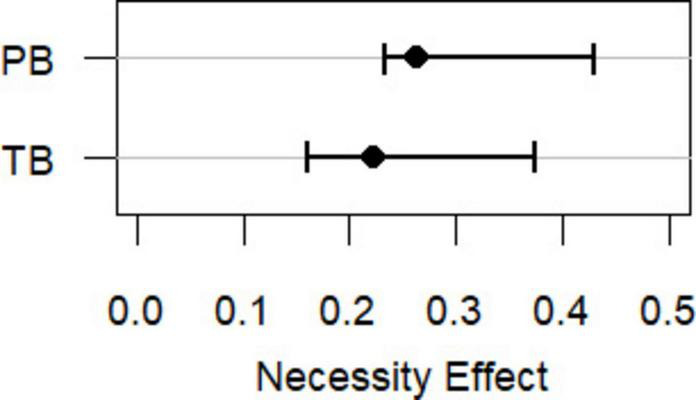
Necessity effects of perceived burdensomeness (PB) and thwarted belonging (TB) on suicidal ideation (the solid dots) as well as ranges of spuriousness, i.e., 95% confidence intervals of necessity effects that can be expected due to the correlation between the variables (the whiskers).

We inserted the sample size (*N* = 340) and correlations between suicidal ideation and perceived burdensomeness (*r* = 0.75) and thwarted belonging (*r* = 0.57) into the NCArigour function. The function generated 1000 datasets with two continuous variables with a bivariate normal distribution and the required correlation between the variables and sample size. The necessity effect (CE-FDH) was estimated in each of these 1000 datasets and a non-parametric 95% percentile confidence interval (i.e., a range of spuriousness) across the 1000 values. Analyses indicated that necessity effects of perceived burdensomeness and thwarted belonging on suicidal ideation (*d* = 0.26 and *d* = 0.22, respectively) fell within the range of spuriousness (0.23–0.43 for perceived burdensomeness and 0.16–0.37 for thwarted belonging, respectively, Figure 1), meaning that they were not significantly stronger than could be expected due to correlations between the variables. This means that the correlations could account for the necessity effects.

**FIGURE 2:**
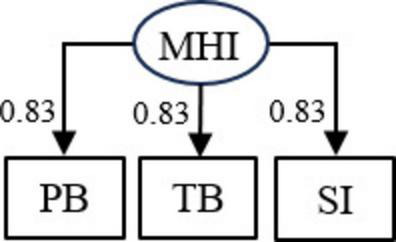
An alternative data generating model where perceived burdensomeness (PB), thwarted belonging (TB), and suicidal ideation (SI) are indicators of general mental health issues (MHI) but have no direct effects on one another. The factor loadings (standardized) were constrained to equality. The model had good fit (χ^2^ = 14.4, *DF* = 5, *p* = 0.013, CFI = 0.980, TLI = 0.988, RMSEA = 0.074 [90% CI: 0.031; 0.121]).

As an alternative to conclusions by Marchetti et al. that perceived burdensomeness and thwarted belonging are necessary for suicidal ideation, we propose that data may have been generated by the model in Figure 2. Here, perceived burdensomeness, thwarted belonging, and suicidal ideation are indicators of a latent mental health issues variable, but the indicators have no direct effects on one another. The model in Figure 2 had good fit even when constraining the factor loadings to equality (χ^2^ = 14.4, *DF* = 5, *p* = 0.013, CFI = 0.980, TLI = 0.988, RMSEA = 0.074 [90% CI: 0.031; 0.121]). It is important to note that data generation in accordance with the model in Figure 2 would be sufficient to generate the necessity effects reported by Merchatti et al., i.e., no additional conditions, e.g., direct effects between the observed variables, would need to be fulfilled.

The model in Figure 2 indicates that individuals with low scores on perceived burdensomeness and thwarted belonging may not, as suggested by Marchetti et al., be immune to suicidal ideation. Would general mental health issues increase in severity, we could expect an increase in all three indicators. The suggestion by Marchetti et al. appears to assume that individual’s perceived burdensomeness and thwarted belonging may never change. As an analogy, this would be like concluding that locations where the sky is blue at a specific point in time will never experience rain. In our opinion, characterizing individuals with low scores on measures of perceived burdensomeness and thwarted belonging at a specific point in time as immune to suicidal ideation would be a potentially hazardous oversimplification of complex psychological phenomena.

## Summary and concluding remarks

Marchetti et al. recommended researchers of mental health to use necessary condition analysis (NCA). In an empirical application, they found statistically significant necessity effects of perceived burdensomeness and thwarted belonging on suicidal ideation. However, necessity effects in NCA do not prove that X is necessary for Y, as the effect could, for example, be due to a correlation between X and Y and this correlation could, in turn, be due to a confounding impact by a third variable Z. A correlation between X and Y is sufficient for a necessity effect of X on Y in NCA, which is unfortunate for a method that claims to assess associations that are fundamentally different from correlations. It is important for researchers using NCA to be aware of this limitation of the method.

It should be noted that we do not necessarily advocate complete abandonment of NCA. Estimating the size of the (semi) empty space in the upper-left corner in a XY-plot may, just like zero-order correlations, have some descriptive value. However, a correlation between X and Y does not prove causality unless alternative explanations, e.g., confounding by a third variable Z, are ruled out. Likewise, a necessity effect of X on Y in NCA does not prove a genuine necessity effect unless ruling out alternative explanations, e.g., that the necessity effect is due to a correlation between X and Y. Therefore, we recommend researchers to scrutinize findings by estimating ranges of spuriousness and to require that necessity effects are above this range if concluding that X is necessary for Y. Otherwise, the necessity effect is not significantly stronger than could be expected due to the correlation between X and Y. The NCArigour function, available in our analytic script at https://osf.io/n7gsk/, can be used to evaluate claimed necessity effects. Conclusions by Marchetti et al., that perceived burdensomeness and thwarted belonging are necessary for suicidal ideation, did not survive scrutiny by this method and may, therefore, be challenged.

## Data Availability

Publicly available datasets were analyzed in this study. This data can be found here: the analytic script, which also downloads used data, is available at the Open Science Framework at https://osf.io/n7gsk/.
